# Geostress in the Ordovician system of the northern segment of the Shunbei No. 4 fault zone based on finite element simulation

**DOI:** 10.1038/s41598-025-03844-5

**Published:** 2025-06-04

**Authors:** Xingqiang Feng, Zhuliang Qian, Lei Zhou, Shaowei Huo, Jun Liu

**Affiliations:** 1 Institute of Geomechanics, Chinese Academy of Geological Sciences, No. 11 Nanminzu Road, Haidian District, Beijing, 100081 China; 2 Key Lab of Petroleum Geomechanics, China Geological Survey, Beijing, 100081 China; 3 Geophysical Research Institute of Jiangsu Oilfield, Sinopec, Nanjing, 210000 China; 4 College of Resource and Environment, Yangtze University, Wuhan, 430100 China; 5 Research Institute of Petroleum Exploration and Development, Northwest Oilfield Company, SINOPEC, Urumqi, 830011 Xinjiang China

**Keywords:** Adaptive finite element analysis (AFEA) numerical simulation, Shunbei fault zone No. 4, Northern segment, Ordovician, Geostress analysis, Solid Earth sciences, Crude oil

## Abstract

The principal focus of geostress studies in the Shunbei area has been on stress measurements, one-dimensional stress profiles, and the stress distribution across planes. However, research on the three-dimensional (3-D) geostress field of the Ordovician system is relatively lacking, and investigations into the stress states of faults have not yet been conducted, thereby hindering the progress of oil and gas exploration and development in this area. This article comprehensively utilizes geological, logging, and seismic data to establish a high-resolution 3-D structural geological model. Based on the results of rock mechanics tests on core samples, acoustic logging data, and 3-D pre-stack inversion datasets, a rock mechanics model for the study area is constructed. In addition, employing finite element simulation methods, the present-day geostress field of the Ordovician system in the study area is numerically simulated, thereby establishing the 3-D geostress field and elucidating the distribution characteristics of the Ordovician system’s geostresses. It systematically analyzes the stress state and activity of the strike-slip faults, highlighting their impact on drilling productivity. Our results provide a basis for the selection of drilling targets and well site deployment. The results lay a foundation for deepening the understanding of the interaction mechanisms between the geological structures and underground stress fields, guiding petroleum resource development, and promoting related geological scientific research in the Ordovician system or geostress mechanics.

## Introduction

The geostress is the sum of the stresses within the Earth’s crust, and the magnitude of the stress depends on the depth, pore pressure, and geological movements on various temporal and spatial scales^1^. The geostress is a crucial factor controlling the formation of oil and gas reservoirs. It governs the types and evolutionary processes of hydrocarbon-bearing basins, as well as the generation, development, and combination rules of basin structures in space and time^2^. It also influences the migration and accumulation of oil and gas. The current distribution of the geostress has significant implications for oil and gas exploration and development. The present-day stress state is pivotal for fault critical stress analysis in oilfields, especially ultra-deep ones, as well as in the design of drilling trajectories, deployment of well networks, and optimization of fracturing operations.

Reservoirs in the Ordovician system in the study area are fault-controlled that are dominated by major strike-slip faults, and the distribution of the hydrocarbon reservoirs is closely related to the deep-seated strike-slip faults. The stress state of these faults directly impacts their activity, leading to variations in the hydrocarbon enrichment and drilling productivity. Given the vital role of geostress in the exploration and development of oil and gas in this region, previous studies have conducted geostress investigations. Employing methods such as anelastic strain recovery and drilling-induced tensile fractures, the stress conditions at a depth of 7 km in the Tarim Basin were analyzed^3^. In the Santangmu Formation in the Ordovician system in the Shunbei Oilfield, weak bedding planes in the dolerite layers have been found to cause borehole failure and collapse due to stress-induced wellbore instability^4^. The Yijianfang-Yingshan Formation in the same system hosts fault-fractured reservoirs influenced by strike-slip faults, forming zones of stress concentration with a substantial horizontal stress differential^5–7^. Based on rock mechanics experiments and array acoustic logging data, a one-dimensional geostress profile of the Ordovician in the fourth zone of the Shunbei was established^8^. Using the ANSYS software for numerical stress field simulation, the planar distribution of the stress in the Yijianfang Formation has been investigated based on rock mechanics experimentation^9^. The in-situ stress states, the fracture systems around faults, and the well productivity characteristics in different segments of the Shunbei No. 4 fault zone were analyzed by using geomechanical theories in the Ordovician system^10^. Significant variations in internal stress states in the Ordovician system were observed across different segments during periods of strike-slip fault activity regulated by multi-stage structural stress^11^. Overall, research in the Shunbei area has primarily focused on stress measurements, one-dimensional stress profiles, and the planar stress distributions,however, there is a relative dearth of research on the three-dimensional (3-D geostress field of strike-slip fault zone, and no studies have specifically addressed the stress state of the faults, hindering the progress of oil and gas exploration and development in this region.

High-precision structural geological model and rock mechanics parameter model are the foundation for ensuring the accuracy of the geostress model. To address this research gap, we utilize geological, logging, and seismic data to construct a high-resolution 3-D structural geological model. Rock mechanics models for the study area are established using the results of rock mechanics tests on core samples, sonic logging data, and 3-D pre-stack inversion datasets. Building upon the results, finite element modeling is employed to numerically simulate the present geostress field of the Ordovician system in the study area, thereby establishing a 3-D geostress field. This work elucidates the distribution characteristics of the geostress in the Ordovician system, systematically analyzes the stress state and activity of the strike-slip faults and highlights their impact on drilling productivity. The results lay a foundation for deepening the understanding of the interaction mechanisms between geological structures and underground stress fields, guiding petroleum resource development, and promoting geological scientific research.

## Overview of the study area

**Fig. 1:**
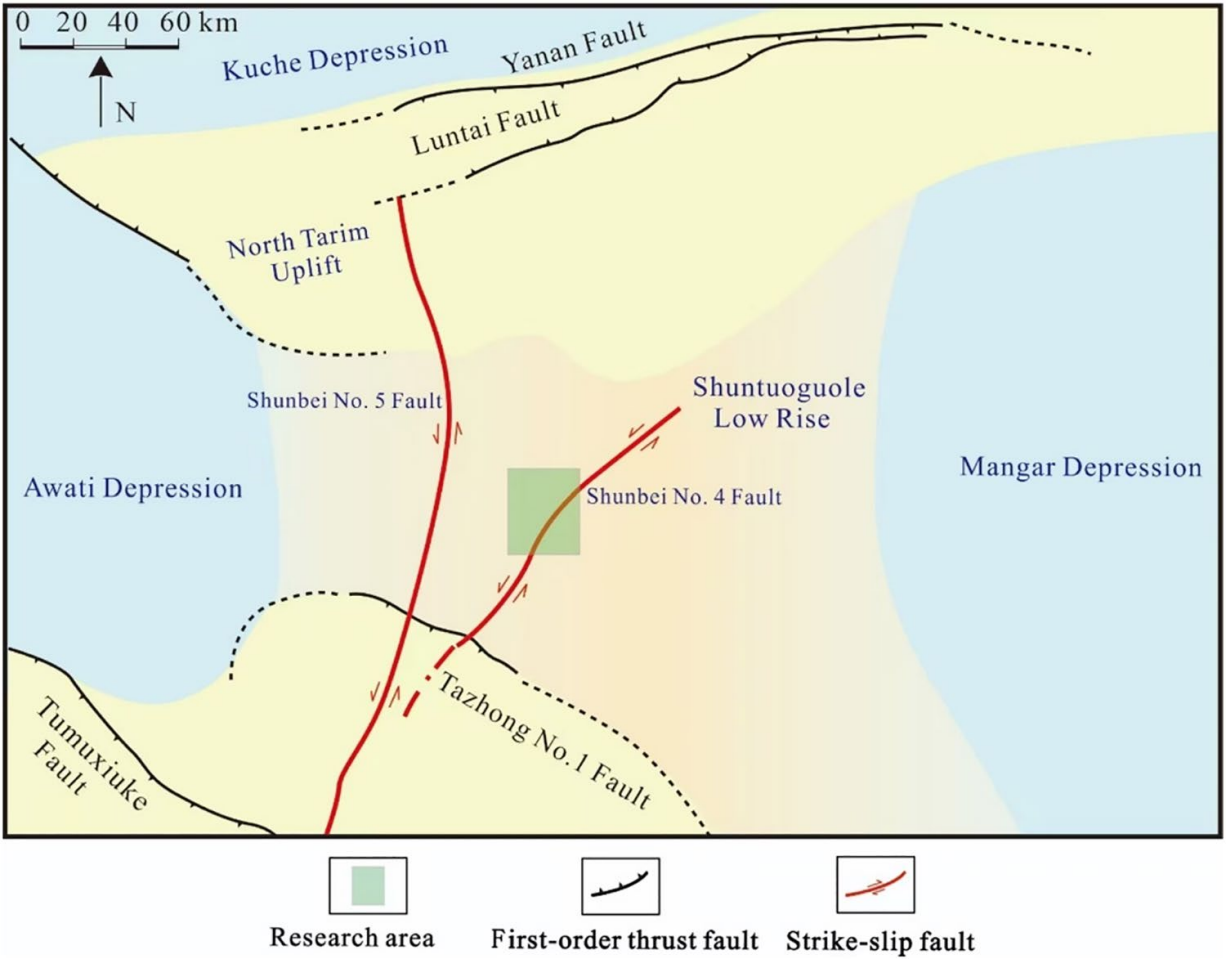
Location of the research area.

The study area is located in the Tarim Basin, Xinjiang, China.The Tarim Basin, located between the Tianshan and Kunlun mountain folds, is China’s largest inland superimposed and composite oil and gas-bearing basin. The study area is situated in the central part of the Tarim Basin, specifically in the Shuntuoguole area, covering approximately 580 square kilometers. The Shuntuoguole region is located amidst the Tabei uplift, Tazhong Uplift, Mangar Depression, and Awati Depression and has undergone multiple tectonic movements including the Caledonian, Hercynian, Indosinian, and Himalayan orogenies (Fig. 1).

Recent oil and gas discoveries in the Shuntuoguole region have revealed its extensive hydrocarbon-bearing pattern, with approximately 18° to the northeast-trending strike-slip fault zones controlling an area of about 34,000 km^2^. It contains estimated oil and gas resources amounting to 1.7 billion tons, comprised of 1.2 billion tons of oil and 500 billion cubic meters of natural gas.

In the study area, from bottom to top, the Ordovician system is comprised of the Lower-Middle Ordovician Yingshan Formation, Middle Ordovician Yijianfang Formation, and the Upper Ordovician Qarabulak Formation, Lianglitage Formation, and Sangtangmu Formation. Among these, the Yijianfang and Yingshan formations are the primary targets for exploration. The Shunbei No. 4 Fault cuts multiple tectonic units and extends northward to the eastern flank of the Tabei Uplift and southward to obliquely intersect with the Tazhong No. 1 Fault in the Tazhong Uplift. Within the study area, the Shunbei No. 4 Fault extends for 60 km and exhibits an S-shaped curved trace. Its orientation shifts from south to north, varying from NE48° to NE28° and then back to NE42°. Based on these directional changes, the Shunbei No. 4 Fault can be segmented into three strike sections: northern, central, and southern. The main focus of the study area lies within the northern segment of the Shunbei No. 4 fault zone.

## Adaptive finite element analysis (AFEA) numerical simulation and stress field simulation process

### Introduction to AFEA numerical simulation

Finite element analysis (FEA) is a numerical computational method used to solve various complex problems in engineering and physics, such as structural mechanics, heat conduction, and fluid mechanics. In FEA, the actual engineering or physical problem needs to be first transformed into a mathematical model to determine the geometry, boundary conditions, material properties, and external loads of the problem. The continuous physical model is discretized into a finite number of subdomains or elements, which can have different shapes such as triangles, quadrilaterals, tetrahedra, or hexahedra. The field quantities within each element are approximated by mathematical functions^12^. By applying force balance and displacement continuity conditions to each element, a system of algebraic equations for the entire system is formed. These equations are usually derived from material constitutive relationships, motion equations, and boundary conditions. Then, the algebraic equations formed are solved using numerical methods to obtain the field quantities at each node. Post-processing of the solution includes computing various physical quantities of interest and performing visualization and result analysis^13^.

AFEA numerical simulation is a commonly used method in the field of engineering technology. It is a form of finite element analysis, which is a numerical computational technique used to solve various engineering problems such as structural analysis, heat conduction, and fluid mechanics. AFEA specifically refers to FEA that employs adaptive techniques^14^. In traditional FEA, the mesh is typically fixed, while in AFEA, the mesh can be automatically adjusted according to the requirements and characteristics of the problem. This allows for more efficient use of computational resources, reduces the computation time, and improves the accuracy of the results. AFEA is commonly used for engineering problems that require high precision and efficiency, such as stress analysis of complex structures and studying the deformation behavior of materials. (Full name of software and Version number in this paper:VISAGE version 2022.4. URL link for VISAGE: https://www.slb.com/products-and-services/delivering-digital-at-scale/software/visage).

### Stress field simulation process

**Fig. 2:**
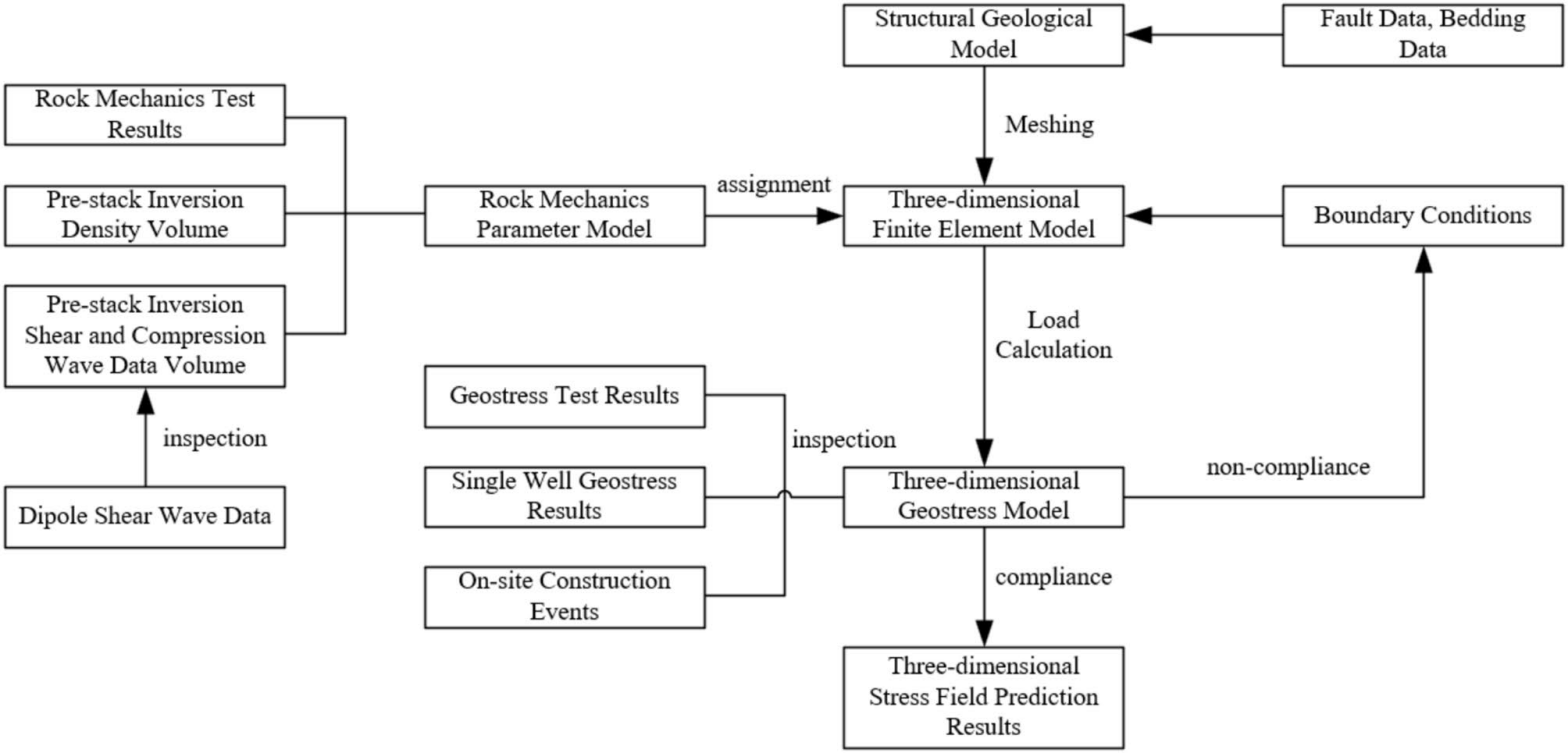
Simulation process of the geostress field of the Ordovician system in the northern segment of the Shunbei fault zone based on adaptive finite element analysis (AFEA) numerical simulation.

The simulation process of the geostress field of the Ordovician system in the northern section of the Shunbei fault zone based on AFEA numerical simulation is illustrated in Fig. 2. First, it is necessary to collect information on the geological structures, formation thickness, and lithology for the study area and to use this information to establish a 3-D geological mechanics model, including modeling of the geological structures, establishment of the formation distribution, and geometric description of the fault zones^15–17^. By integrating rock mechanical parameter measurements, pre-stack inversion P-wave and S-wave velocities, and density data, a 3D rock mechanical parameter model was constructed. Based on the actual geological conditions and previous research results, the boundary conditions for the simulation are determined, including the boundaries of the simulation area, external forces applied, and displacement or fixed constraints at the boundaries. In simulating the geostress field, the finite element method or finite difference method is commonly used. When choosing the appropriate numerical simulation method, factors such as the model complexity, computational efficiency, and simulation accuracy need to be considered. The geological model is converted into a numerical model and is grid partitioned. In the fault zone area, finer grid partitioning may be required to capture the geometric features and mechanical behavior of the fault zone^18^. Boundary conditions are applied, and gravity is loaded onto the finite element cells at the same time. Using AFEA software, the established geological mechanics model is numerically simulated, and the distribution of the geostress field within the simulation area is calculated. The numerical simulation results are analyzed, including the magnitude, directional distribution, stress concentration areas, and other features of the geostress field. Based on the simulation results, a deeper understanding of the geostress state in the fault zone area can be obtained, providing references for subsequent geological engineering design and development^19^. The model parameters and boundary conditions are evaluated based on the simulation results, and adjustments are made to improve the accuracy and reliability of the simulation results. Based on adaptive finite element analysis, the simulation process for the geostress field of the Ordovician system in the northern segment of the Shunbei fault zone has been established (Fig. 2).

## Modeling of 3-D structural geology and finite element model

### Three-dimensional structural geology modeling

The generation of fault surfaces requires the calculation and generation of fault planes using certain interpolation methods. Among them, the kriging interpolation method has been widely used in fields such as geology, geographic information systems, and environmental science. The kriging interpolation method is based on the spatial correlation between surface data points, so it can more accurately reflect the spatial distribution pattern of surface data^20^. It is particularly suitable for situations where surface attribute values have spatial trends or strong spatial autocorrelation. The kriging interpolation method estimates the values of unknown points by fitting a semivariogram function. Smooth and continuous surfaces are typically generated in the interpolation results, making them more consistent with the actual surface features and facilitating the analysis and prediction of the terrain, geology, and other attributes. The kriging interpolation method can estimate the uncertainty of each interpolation point, i.e., the confidence of the predicted value, which is crucial for determining the reliability and credibility of the interpolation results, thus helping users better understand the interpolation results and to make rational decisions. It is not limited by the regularity of the data point distribution and is suitable for application to irregularly distributed surface data. It can adapt to different data point distributions by selecting appropriate semivariogram functions, thereby obtaining more accurate interpolation results. The principle of the kriging interpolation method is relatively simple, and the parameter settings are relatively intuitive^21^. It usually only requires the coordinates and attribute values of the data points as inputs, without additional complex parameters or assumptions. Therefore, the kriging interpolation method has a high operability and ease of use in practical applications.

**Fig. 3:**
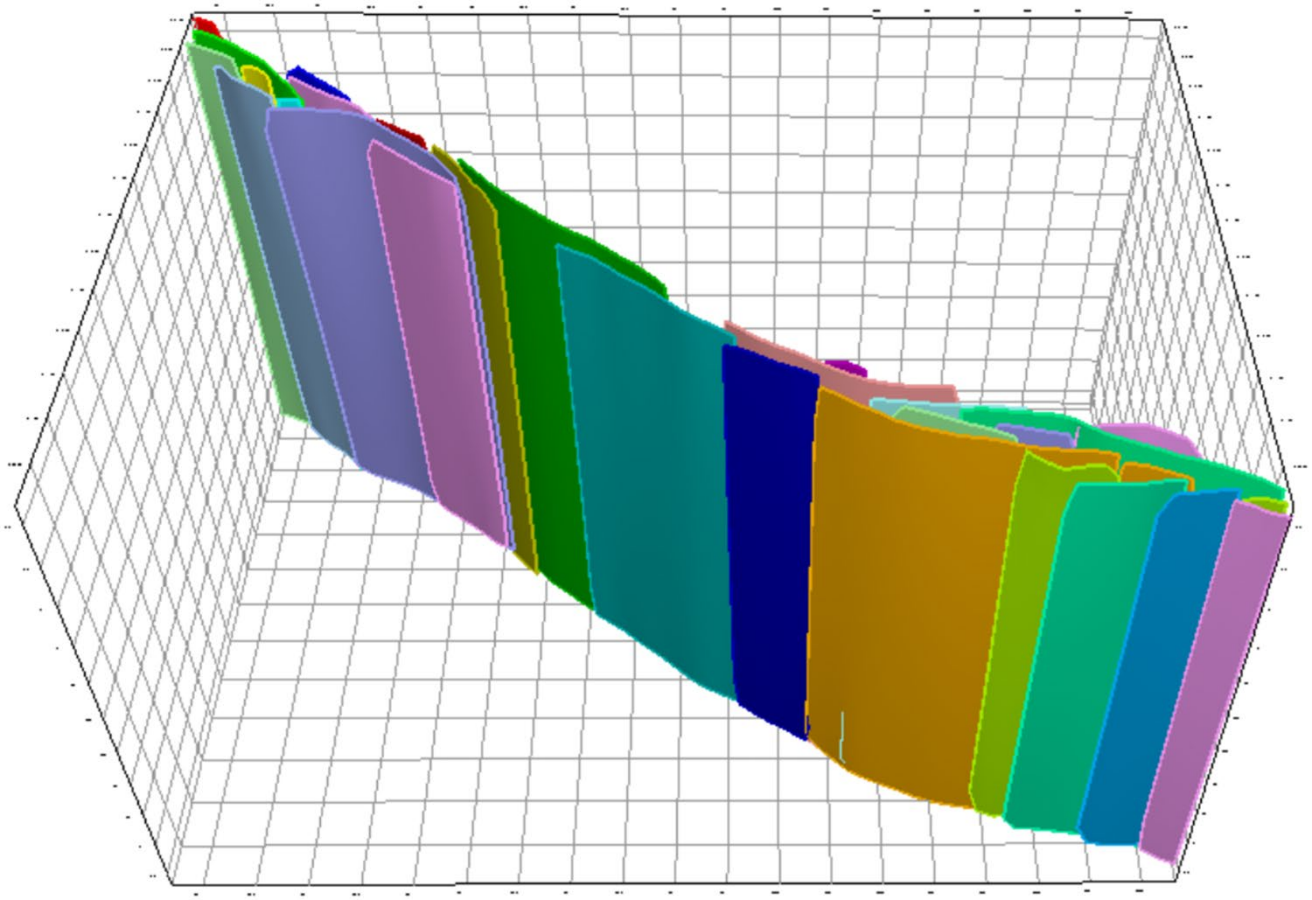
Fault model generated via kriging interpolation.

Based on the advantages of the kriging interpolation method, in this paper, this method is adopted to form the fault surfaces. The resulting fault model is shown in Fig. 3.

**Fig. 4:**
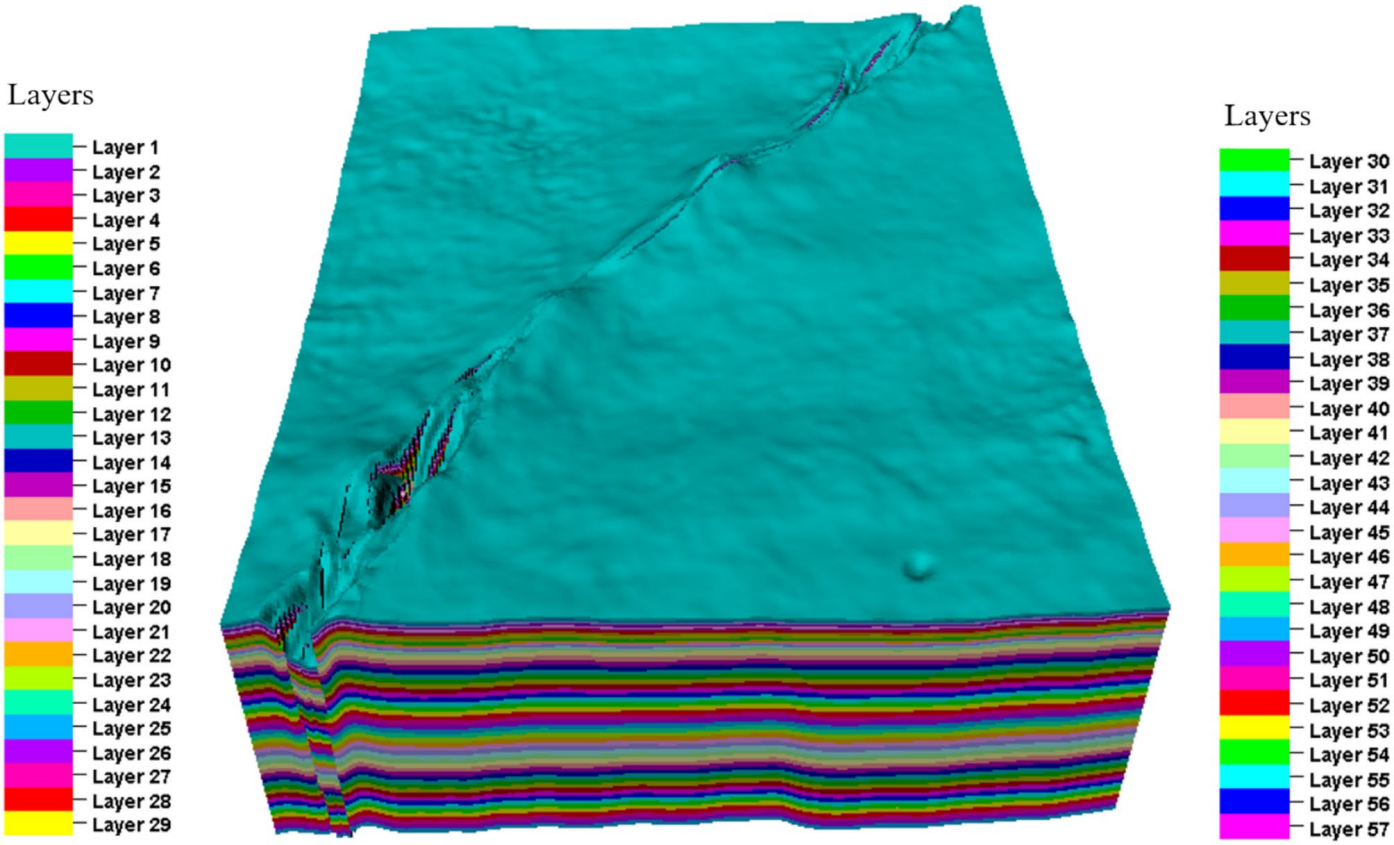
Stratigraphic model generated via kriging interpolation.

In this study, the modeling data for the key layers mainly included seismic layer data and well layer data. On this basis, three profiles are randomly selected for observation. In the T_7_^4^, T_7_^5^, and T_7_^8^ layers, there was good consistency with the fault model vertically. Therefore, the requirements for layering were relatively small. The target layer mainly consists of micritic limestone and sand-clastic micritic limestone. To account for the impact of lithology on rock mechanical properties, the model was vertically layered based on the minimum lithologic thickness observed in the well. Taking T_7_^4^–T_7_^5^ as an example, the minimum response thickness is 8 m, which was divided into 15 layers; while the minimum response thickness of T_7_^5^–T_7_^8^ is 20 m, which was divided into 42 layers. In total, it was divided into 57 layers. Finally, the stratigraphic model was established (Fig. 4).

**Fig. 5:**
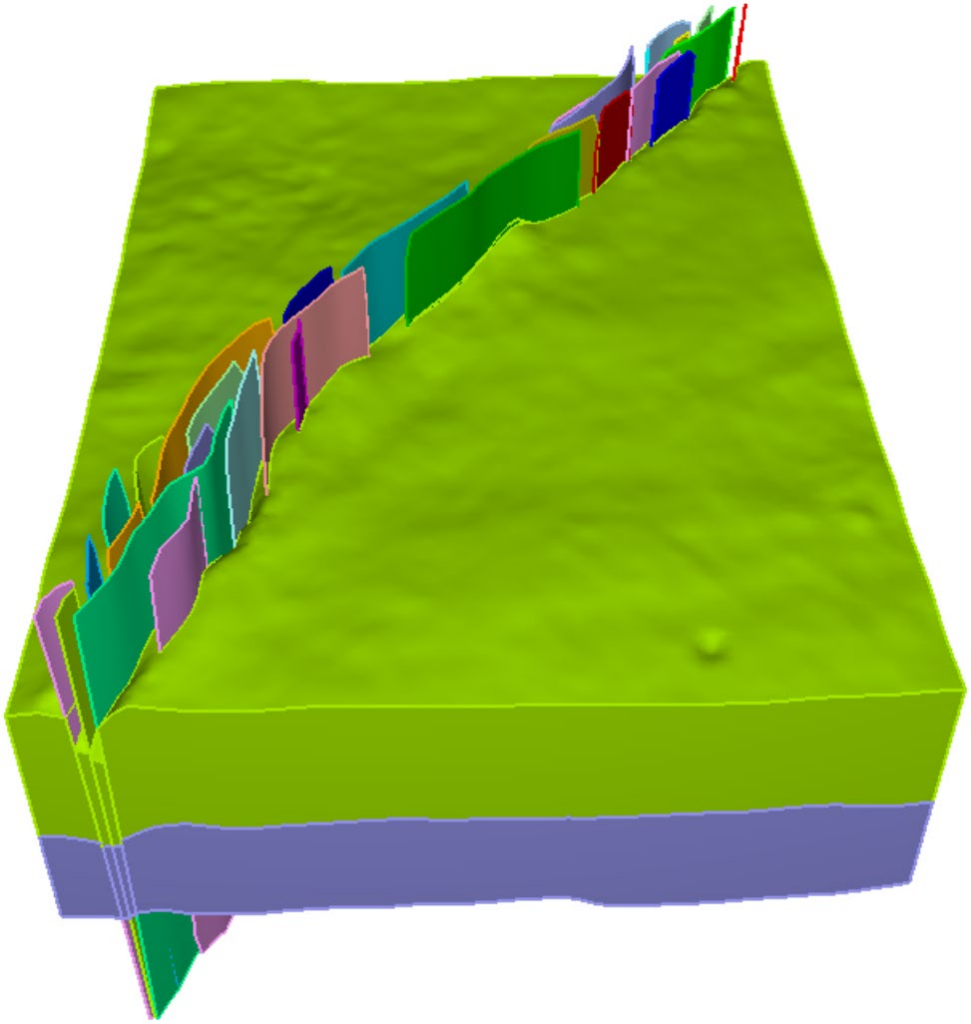
Three-dimensional structural geological model of the target layer in the study area.

During the geological modeling process^22^, first, the combination of the target layer stratigraphy and faults in the study area was sorted based on the fault model and layer model. Through analysis of these combination characteristics, the combination characteristics of the faults and stratigraphy were clearly determined. Based on the clarification of the combination characteristics, a 3-D structural geological model of the target layer in the study area was further established (Fig. 5). This model was established based on considering the combination characteristics of the target layer and faults aiming to more accurately reflect the geological structure and tectonic characteristics of the target layer in the study area.

**Fig. 6:**
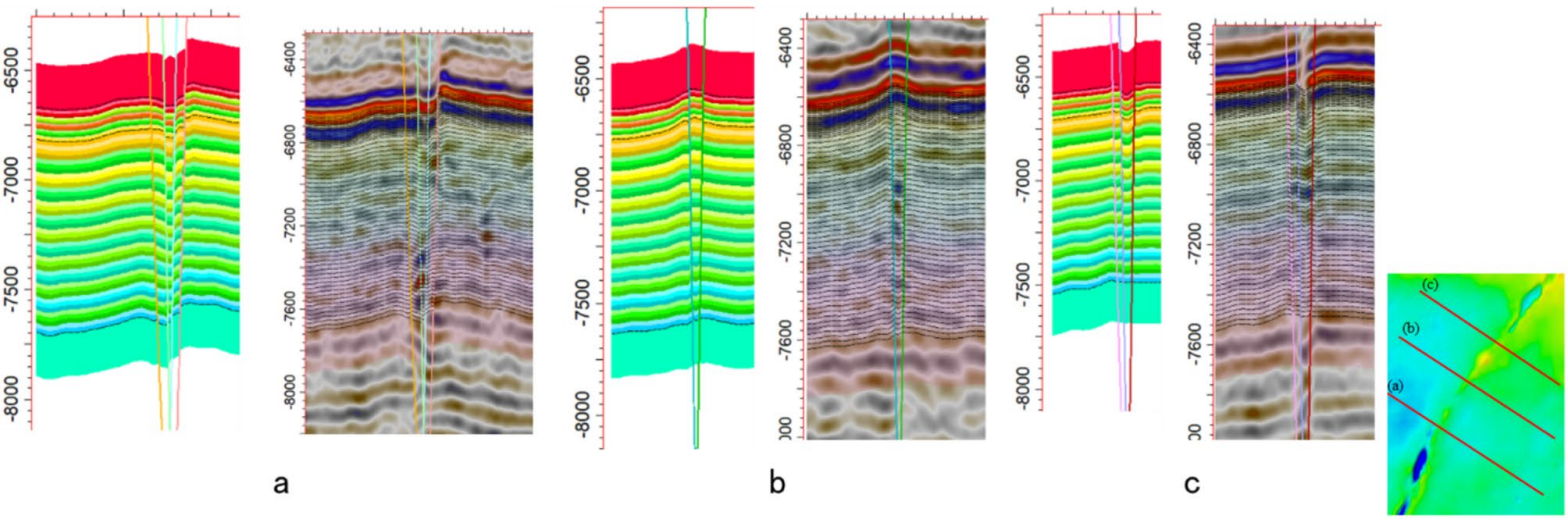
Comparison between model cross-sections and seismic interpretation cross-sections.

An accurate 3D geological model is the foundation for subsequent geostress modeling. To verify the accuracy of the geological model, three random cross-sections were extracted and compared with the seismic interpretation sections used for modeling (Fig. 6). The results show that the stratigraphic layers, fault distributions, and their configurations in the model sections are entirely consistent with the seismic interpretations, indicating that the geological model accurately reflects the subsurface geological characteristics of the study area.

### Three-dimensional finite element modeling

Initially, based on the shape and physical characteristics of the target layer in the study area, the finite element preprocessing modeling method was used to define the boundaries of the 3-D geological model. Adaptive mesh partitioning methods were employed to conduct finite element grid division. This process involves numbering and establishing the relationships between the computational units and nodes. It also requires representing the coordinates of the nodes and listing the node numbers of the natural and essential boundaries and their corresponding boundary values^23,24^. During the unit division process, to balance the accuracy of the calculation results and computational complexity, especially to highlight the stress characteristics of the critical areas, and considering the limitations of the computational capabilities, the fault zone of the target layer was divided into small triangular elements, while the auxiliary model was divided into larger grid elements. Specifically, the research area surrounding the fault zone was divided into finer meshes, while the surrounding rock zone of the fault zone was divided into coarser meshes.

### Rock mechanics parameter model

**Fig. 7:**
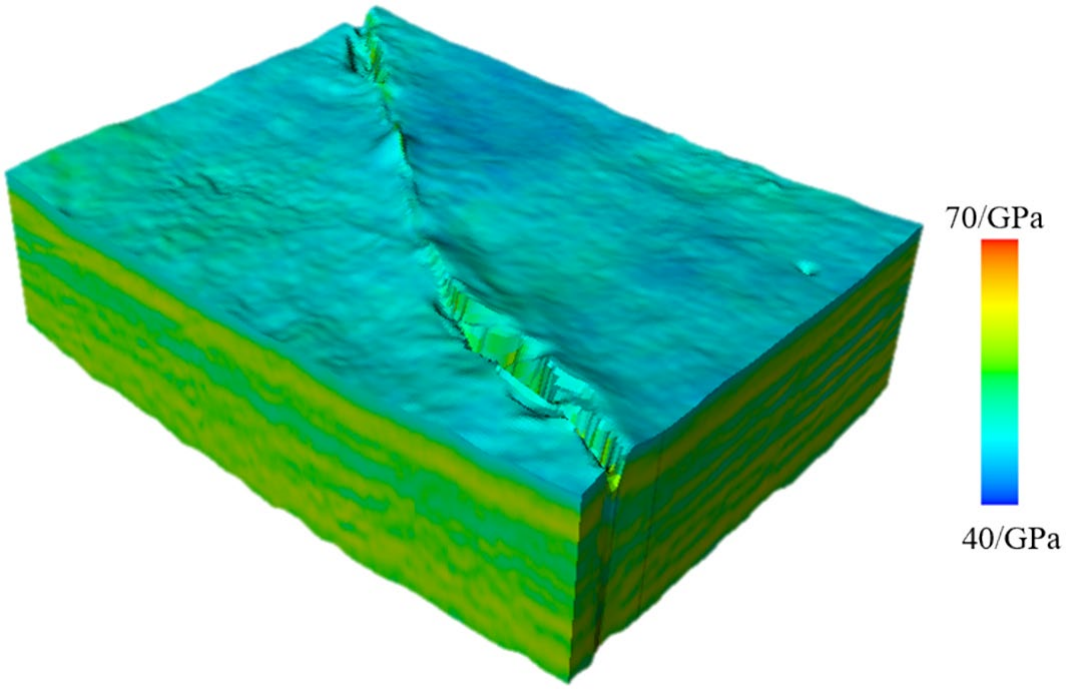
Three-dimensional Young’s modulus model of the target layer in the study area.

**Fig. 8:**
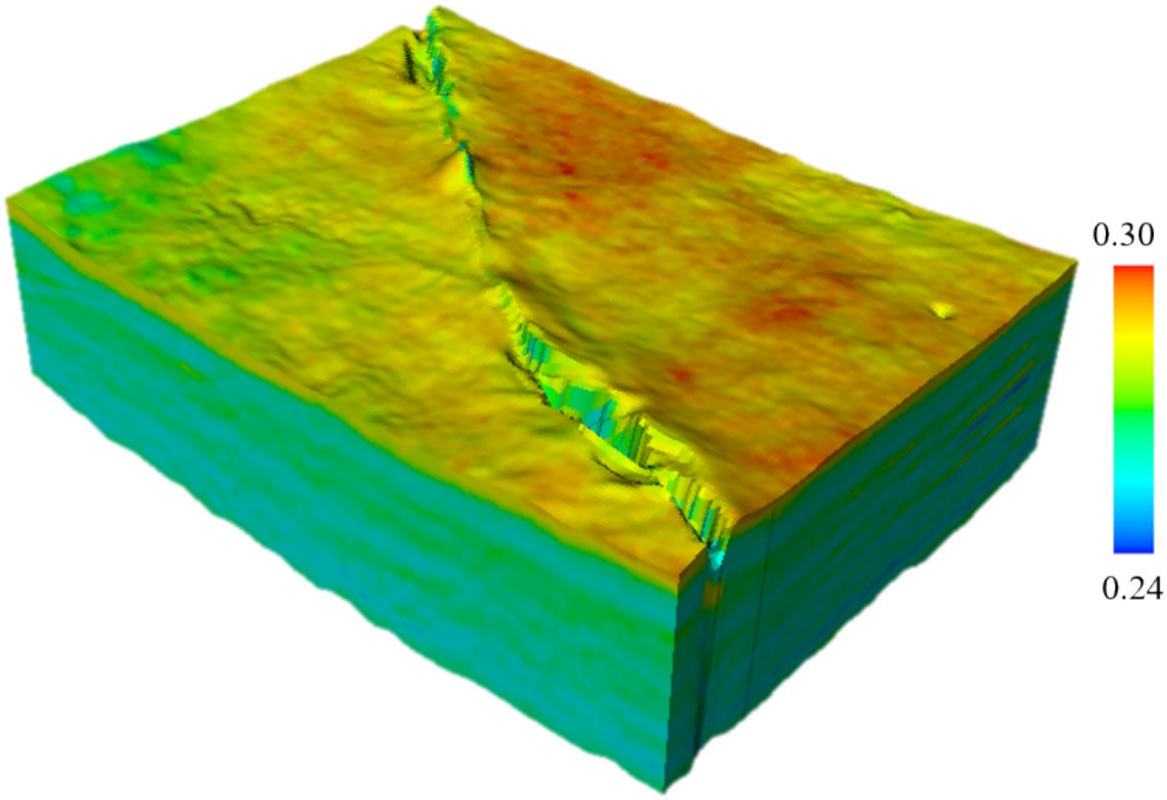
Three-dimensional Poisson’s ratio model of the target layer in the study area.

A rock mechanics parameter model was essential for conducting the finite element numerical simulations. The primary parameters used in this simulation included key elastic properties such as Young’s modulus and Poisson’s ratio. According to the shape of the target layer’s solution domain within the study area and the physical characteristics of the actual problem, the region was subdivided into numerous interconnected, non-overlapping elements. Decomposing the geographical domain was preparatory work for the finite element method, which is substantial. In addition to numbering the computational elements and nodes and determining their relationships, it was necessary to specify the coordinates of the nodal points. In this context, a list was required detailing the node numbers and their corresponding domains and boundary values for significant boundaries. Utilizing finite element preprocessing modeling, with the boundaries defined by the scope of the seismic data encompassing the 3-D geological model, adaptive mesh partitioning was employed to conduct the finite element grid discretization. Static rock mechanics parameters derived from rock mechanics experiments on core samples, in conjunction with dynamic parameters inferred from logging data, were utilized to establish the correlation between the static and dynamic rock mechanics parameters. A 3-D dynamic rock mechanics parameter volume was constructed based on the 3-D seismic inversion data volume. Leveraging the established static-dynamic relationship, 3-D models of the Young’s modulus (Fig. 7) and Poisson’s ratio (Fig. 8) were then formulated. Parameter values were assigned to the finite elements using mesh coarsening methods for reasonable application in finite element grid models^25,26^.

### Setting the boundary conditions

Boundary conditions are a critical factor affecting the accuracy of stress field numerical simulations. First, the boundary conditions were determined based on the tectonic background of the study area. The finite element numerical simulations were conducted by applying boundary loads and constraints, including stress direction, magnitude, and displacement constraints. Multiple iterations and adjustments were typically required. The stress conditions of specific wells were obtained based on existing oilfield logging data^27,28^. It was assumed that the structural forces on the boundaries had certain numerical values. In the model, the depth direction was set as the Z-axis and the gradient was positive. The X-axis represented the east–west direction, while the Y-axis represented the north–south direction. By continuously adjusting the values of the boundary loads, the stress at the characteristic well points in the simulation calculation model was ensured to be consistent with the actual stress. The direction of the maximum horizontal principal stress in the study area was determined through analysis of shear wave anisotropy data obtained from well logging. In accordance with the aforementioned approach, the boundary conditions for the stress simulation in the study area were defined as follows: the loading in the N125°E direction was 146 MPa; the loading in the N35°E direction was 200 MPa; and the orientation of the maximum principal stress was 35°. Gravity is one of the important factors controlling the 3-D distribution of the geostress. Therefore, gravity loading was also applied to the simulation boundary^29^.

### Quality control of simulation results

**Fig. 9:**
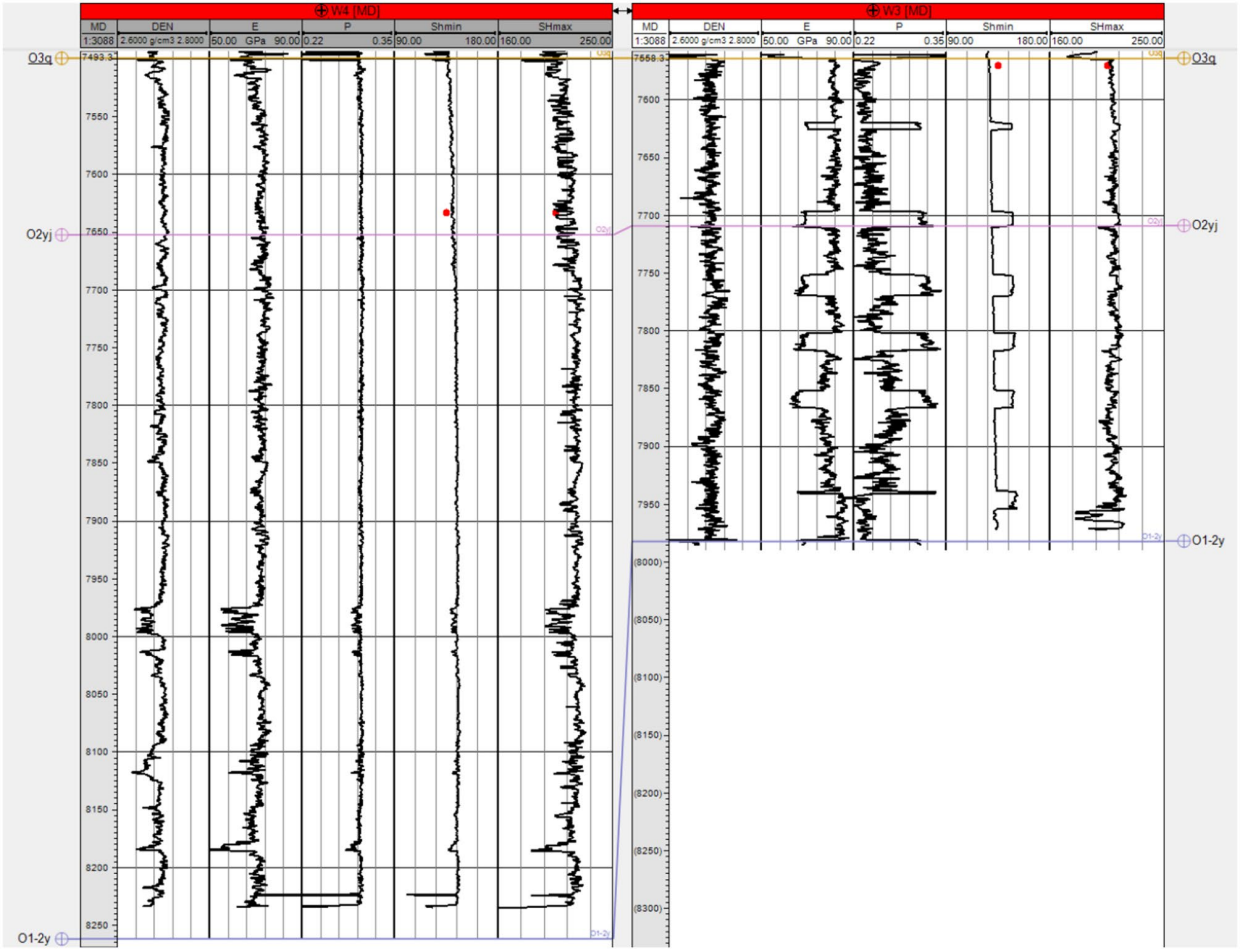
Graph comparing simulated wellbore stress outcomes with experimental outcomes, the red dots signify the experimental wellbore stress results.

During the process of quality control of the simulation results, the geological conditions underground were simulated using geological models and numerical simulation methods. The maximum and minimum horizontal stresses in the simulation results were calculated and compared with the experimental data. The differences and consistencies between them were analyzed, including the numerical values, distribution patterns, and spatial distribution. Based on the comparison of the results, the accuracy and reliability of the geological model were evaluated. The consistency and proximity between the experimental and simulated results indicate that the geological model has a high accuracy. If significant differences exist, further analysis of the limitations and opportunities for improvement of the model is needed. Adjustments to the parameters or structure of the geological model can be considered to the optimal accuracy. The results of the comparison of the experimental data and simulation results are presented in Table 1 (Fig. 9). Through analysis, it was found that the error of the simulation results was relatively small. Therefore, it was concluded that the model established in this study could be used to analyze the stress of the Ordovician system in the northern section of the Shunbei fault zone.

**Table 1:** Comparison of the rock core geostress experiment test data and simulation results.

Well name	W3	W4
Sampling depth (m)	7569.84–7570.05	7632.94–7633.17
Experimental maximum horizontal geostress (MPa)	213	212
Simulated maximum horizontal geostress (MPa)	200	208
Error	6.1%	1.9%
Experimental minimum horizontal geostress (MPa)	143	149
Simulated minimum horizontal geostress (MPa)	150	148
Error	4.6%	0.7%

## Stress analysis of the Ordovician system in the northern segment of the Shunbei fault zone

### Characteristics of stress field distribution on planes

**Fig. 10:**
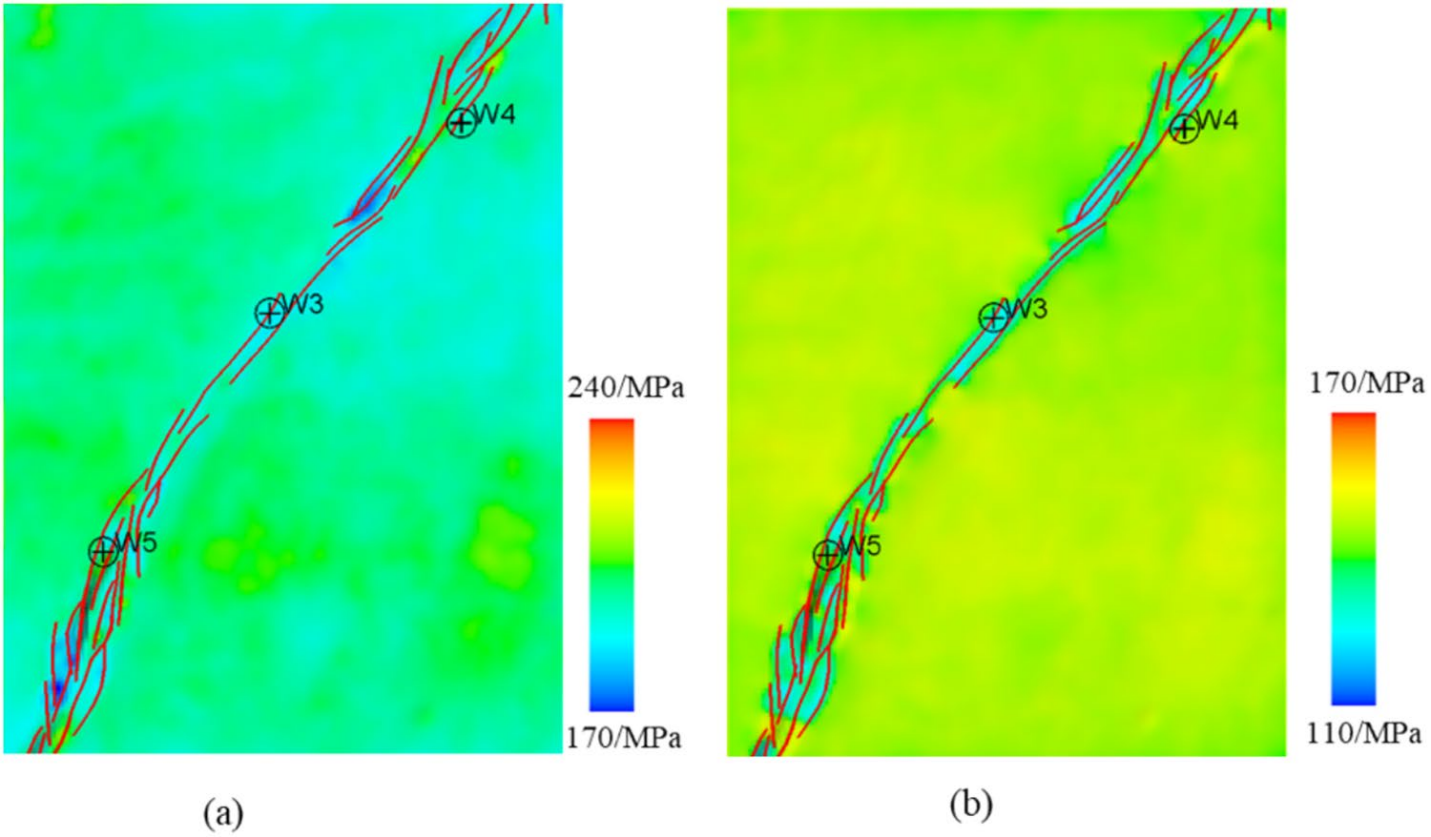
Distribution of stress in the Yijianfang Formation in the study area: (**a**) maximum horizontal principal stress; (**b**) minimum horizontal principal stress.

According to the simulation results, the stress distribution in the target layer in the study area exhibits distinct spatial features. In the northern region, the maximum horizontal principal stress of the Yijianfang Formation exhibits relatively low values, approximately 200–205 MPa, which are lower than those in the high-value areas in the southwestern and central regions (around 230 MPa). The minimum horizontal principal stress values of the Yijianfang Formation in the northern region are 135–143 MPa, which are lower than those in the southwest. Near the fault zone and the adjacent strata, the minimum horizontal principal stress values are lower, approximately 143 MPa, while those of the western strata, which are farther away from the fault zone, are higher, with a maximum value of about 143 MPa (Fig. 10).

**Fig. 11:**
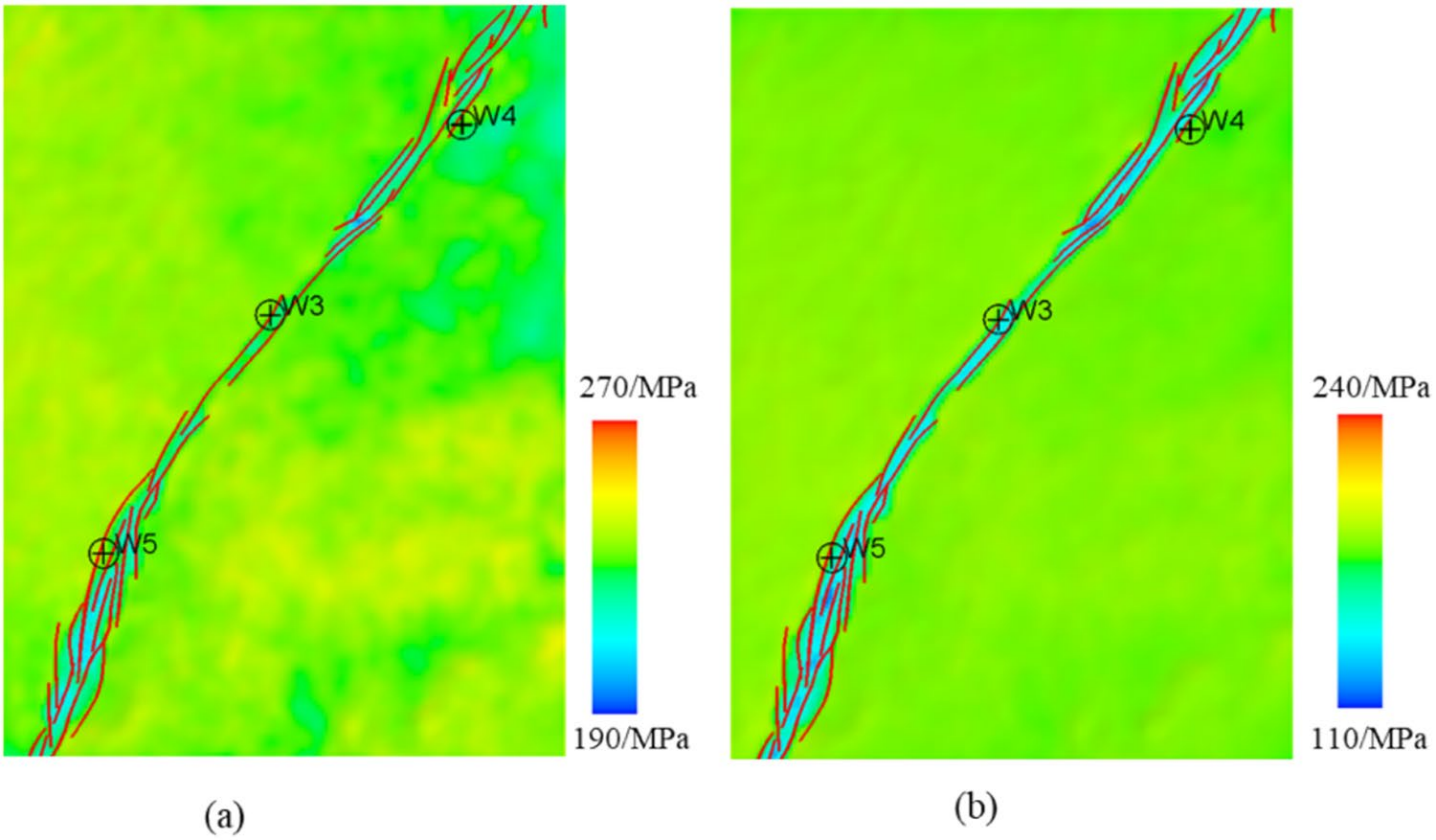
Distribution of stress in the Yingshan Formation in the study area: (**a**) maximum horizontal principal stress; (**b**) minimum horizontal principal stress.

The high-value areas of the maximum horizontal principal stress in the Yingshan Formation are mainly distributed in the southern part of the study area, with values of around 243 MPa. The low-value areas are primarily located in the northeastern part and within the strike-slip fault zone, with values of approximately 219 MPa. Overall, the maximum horizontal principal stress decreases along the fault zone from the southwestern to the northeastern parts of the study area. The minimum horizontal principal stress ranges between 152 and 184 MPa. Within the strike-slip fault zone, the minimum horizontal principal stress values are lower, while the values are higher in strata farther from the fault zone in the west, reaching a maximum value of about 184 MPa in the western strata near the fault zone (Fig. 11).

### Variations in the stress field in the vertical direction

**Fig. 12:**
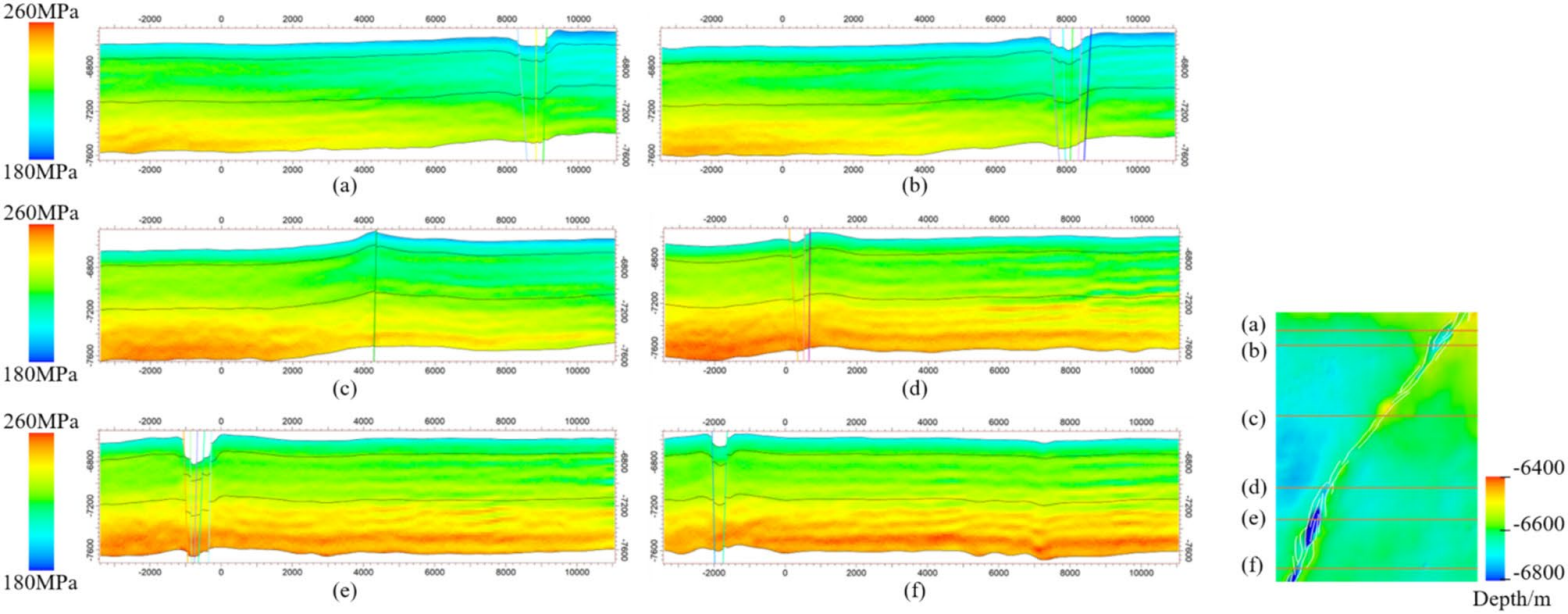
The vertical principal stress section at different positions of the study area’s target layer. (**a** and **b**) the vertical principal stress section at the northern parts of the target layer; (**c** and **d**) the vertical principal stress section at the central parts of the target layer; (**e** and **f**) the vertical principal stress section at the southern parts of the target layer.

According to the simulation results, the variation in the stress field in the target area increases gradually with depth. The western strata in the central and northern parts of the target layer have greater burial depths compared to those of the eastern strata (Fig. 12a–d). The variation in the maximum principal stress in the vertical direction in the western strata gradually decreases towards the east. In contrast, the gradient of the vertical variation in the maximum principal stress in the strata near the fault zone and the area to the east is relatively small. Overall, the western strata near the fault zone exhibit a higher vertical variation in the maximum principal stress, while the gradient in the eastern strata is smaller (Fig. 12a–d). The burial depth of the strata in the eastern and western parts of the fault zone within the southern part of the study area’s target layer does not differ significantly, and the vertical gradient of the maximum principal stress change is consistent (Fig. 12e,f). These characteristics illustrate the influence of the fault zone on the stress field, as well as the differences in the vertical variations in the maximum principal stress in the western and eastern strata. Gravity is the primary factor contributing to the stress generation in the deeper strata. According to the burial depth map, the western strata in the central and northern parts of the target layer have greater burial depths compared to those of the eastern strata.

### Stress characteristics in the fault zone

In the study area, the reservoirs are primarily carbonate rock reservoirs with relatively poor physical properties, making them unsuitable as effective reservoirs. The development of the reservoirs in this region is greatly influenced by the strike-slip fault zones, which cut through the target layers, forming channels and increasing the porosity and permeability to create effective storage spaces. The ratio of the shear stress to the normal stress on a fracture surface is a critical factor controlling the relative sliding on the fracture surface and is an important geological property that governs the permeability of the fracture zone^30–32^. Therefore, by integrating the fractures with the 3-D geostress field models, we analyzed the force characteristics of the different fractures and within each grid element of a fracture, quantitatively determined the ratio of the shear stress to the normal stress in each grid of the fracture body, and determined the critical stress fault using the ratio of the shear stress to the normal stress. This indirectly predicted the relative permeability and flow capacity within the fracture body. Therefore, in this study, we conducted a detailed analysis of the stress states of the fault zones surrounding the drilling sites in the study area in order to investigate the impact of the critical stress faults on the drilling production in the study area.

We utilized the Mohr–Coulomb failure criterion, expressed by Eq. (1), to ascertain if a fault was under its critical stress condition.

1 $$\frac{\tau }{{\sigma }_{n}}=\mu ,$$

where *τ* is the shear stress on the fault surface, ${\sigma }_{n}$ is the effective normal stress; and *μ* is the coefficient of friction. The value of *μ* is generally set to 0.6.

The above equation can be transformed to Eq. (2).

2 $$\text{CFF}=\tau -\mu {\sigma }_{n}.$$

When the value is negative, the fault plane remains stable as the shear stress is insufficient to overcome the frictional resistance. However, when the Coulomb failure function (CFF) reaches zero, on the pre-existing fault plane, the shear stress can overcome the effective normal stress, thus triggering slip.

**Fig. 13:**
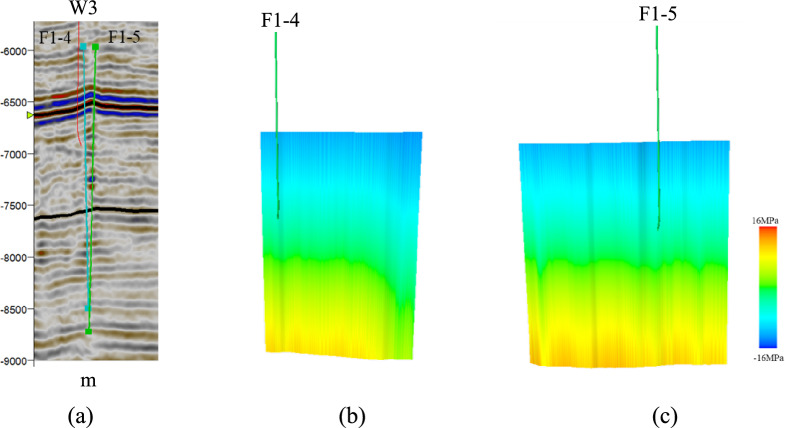
Cross-section of critical stress analysis for the faults near well W3: (**a**) seismic section across well W3; (**b**) critical stress profile for fault F1-4; (**c**) critical stress profile for fault F1-5.

Based on the above equation, critical stress analysis was conducted on the faults near the drilling sites in the study area. In the study area, there are three oil wells: W3, W4, and W5. Well W3 is located in the central part of the fault zone in the study area. It traverses the F1-4 and F1-5 fault zones. Within the F1-4 fault zone, the effective normal stress is 42–85 MPa, and the shear stress is 32–41 MPa. Within the F1-5 fault zone, the effective normal stress is 40–118 MPa, and the shear stress is 30–41 MPa. Based on the normal stress and shear stress on the fault plane, the critical stress profile of the fault was calculated (Fig. 13). The geological conditions in the lower part of the F1-4 and F1-5 fault zones were in a critical state.

**Fig. 14:**
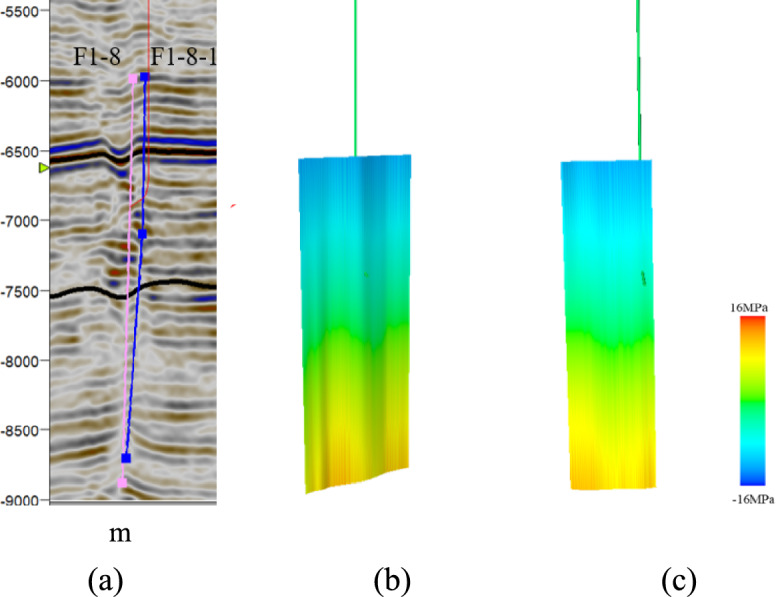
Cross-section of the critical stress analysis for the faults near well W4: (**a**) seismic section across well W4; (**b**) critical stress profile for fault F1-8; (**c**) critical stress profile for fault F1-8-1.

Well W4 is located in the northern part of the fault zone. It traverses the F1-8 and F1-8-1 fault zones. Within the F1-8 fault zone, the effective normal stress is 41–75 MPa, and the shear stress is 32–42 MPa. Within the F1-8-1 fault zone, the effective normal stress is 40–83 MPa, and the shear stress is 35–42 MPa. Based on the normal stress and shear stress on the fault plane, the critical stress profile of the fault was calculated (Fig. 14). The geological conditions in the lower part of the F1-8 and F1-8-1 fault zones are in a critical state.

**Fig. 15:**
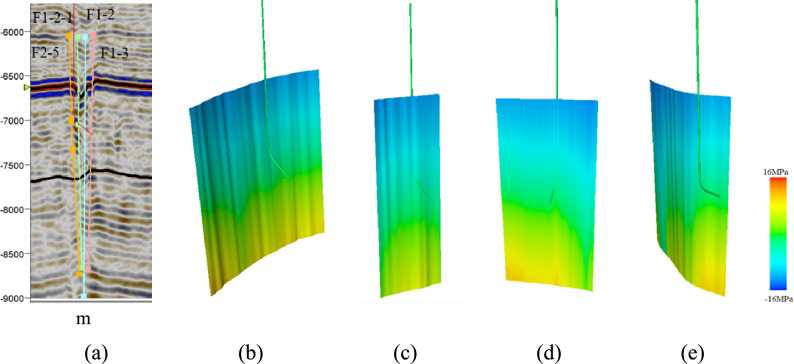
Cross-section of the critical stress analysis for the faults near well W5: (**a**) seismic section across well W5; (**b**) critical stress profile for fault F2-5; (**c**) critical stress profile for fault F1-2-1; (**d**) critical stress profile for fault F1-2; (**e**) critical stress profile for fault F1-3.

Well W5 is located in the southern part of the fault zone. It traverses fault zones F1-2, F1-2-1, and F1-3. Fault zone F2-5 is near Well W5. Within fault zone F1-2, the effective normal stress is 42–114 MPa, and the shear stress is 33–41 MPa. Within fault zone F1-2-1, the effective normal stress is 44–107 MPa, and the shear stress is 35–40 MPa. Within fault zone F1-3, the effective normal stress is 43–138 MPa, and the shear stress is 32–41 MPa. Within fault zone F2-5, the effective normal stress is 42–105 MPa, and the shear stress is 35–40 MPa. Based on the normal stress and shear stress on the fault plane, the critical stress profile of the fault was calculated (Fig. 15). The geological conditions in the lower part of fault zones F1-2, F1-2-1, F1-3, and F2-5 are in a critical state.

There are three wells in the northern segment of the Shunbei fault zone. The relationship between the production of these wells and the nearby faults is presented in Table 2. We observed a correlation between the stress distribution of the faults intersected by the three wells and their production. These faults are in a critical stress state, likely due to the influence of structural forces and gravity on the formations. It can be inferred from experience and observations that a larger number of faults in a critical stress state are generally associated with higher production. This is likely because the faults in a critical state have good connectivity, so they can serve as channels for fluid migration, effectively increasing the porosity and permeability and thereby providing more pathways and storage space for reservoir fluids.

**Table 2:** Relationship between production of two wells and nearby faults.

Well ID	W3	W4	W5
Daily oil production (tons)	759.4	512.9	820.3
Adjacent faults	F1-4, F1-5	F1-8, F1-8-1	F2-5, F1-2-1, F1-2, F1-3
Intersecting faults (number)	2	2	3
Faults in critical state (number)	2	2	4

## Conclusions

In this study, the distribution of geostress in the Ordovician system in the northern section of the Shunbei fault zone was investigated by applying the AFEA numerical simulation method. A geomechanical model was established to investigate the stress distribution. In the northern region, the maximum and minimum horizontal principal stresses of Yijianfang Formation are lower than the stresses in the southwest and central regions. The high-value areas of the maximum and minimum horizontal principal stresses in the Yingshan Formation are mainly distributed in the southern part of the study area. We observed that the carbonate reservoirs in this area are mainly controlled by the strike-slip fault zone in the Shunbei region. The maximum and minimum horizontal principal stresses in this area are generally lower. As the depth increases, the maximum principal stress gradually increases. The western strata near the fault zone exhibit a higher vertical variation gradient of the maximum principal stress, while the gradient is smaller in the eastern strata. By determining the relationship between the stress distribution around three wells and their production, it was found that the higher the number of faults in a critical stress state was, the higher the production was. This indicates that the stress has an impact on the productivity of oil wells.

## Data Availability

Data is provided within the manuscript.

## References

[1] Zoback, M. D. *Reservoir Geomechanics* (Cambridge University Press, New York, 2007). 10.1017/cbo9780511586477.

[2] Ma, Y. The role and significance of crustal stress in petroleum geology and its present situation. *J. Geomech.* **3**(2), 41–46 (1997).

[3] Sun, D. et al. Determination of the in-situ stress state at 7 km depth under Tarim Basin by ASR and DITH methods. *Chin. J. Rock Mech. Eng.* **37**(2), 383–391. 10.13722/j.cnki.jrme.2016.1145 (2018).

[4] Hu, G., Bai, B. & Ke, K. Analysis on borehole instability mechanism of diabase in Shunbei Block. *China Offshore Oil Gas* **29**(5), 119–125. 10.11935/j.issn.1673-1506.2017.05.017 (2017).

[5] Hou, L., Yang, C., Guo, Y., Chang, X. & Wang, L. Simulation study on crack steering of near-wellbore in crack-hole type carbonate rock. *Sci. Technol. Eng.* **20**(27), 11080–11086. 10.3969/j.issn.1671-1815.2020.27.015 (2020).

[6] Wang, W., Li, D., Jin, J., Xu, J. & Zhang, D. Technical problems and measures of wellbore stability of broken formation in Shunbei oil and gas field. *Sci. Technol. Eng.* **22**(13), 5205–5212. 10.3969/j.issn.1671-1815.2022.13.014 (2022).

[7] Zhang, Y., Li, D., Gao, S., Lin, Y. & Zeng, Y. Analysis on influencing factors of wellbore instability of Ordovician fractured formation in Shunbei oil and gas field. *Fault-Block Oil Gas Field* **29**(2), 256–260. 10.6056/dkyqt202202020 (2022).

[8] Liu, J., Huang, C., Zhou, L., Chen, Q. & Zhang, S. Estimation of the rock mechanics and in-situ stress parameters of carbonate reservoirs using array sonic logging: A case study of Shunbei No. 4 block. *J. Geomech.* **30**(3), 394–407. 10.12090/j.issn.1006-6616.2023110 (2024).

[9] Bao, D. et al. Simulation of stress field in ultra deep strike slip fault zone and its development significance: A case study of the southern section of Shunbei No.5 fault zone. *Sci. Technol. Eng.* **23**(31), 13254–13264. 10.3969/j.issn.1671-1815.2023.31.007 (2023).

[10] Huang, C. et al. In-situ stress characteristics and fracture distribution prediction of different segments in Shunbei No. 4 strike-slip fault zone, Tarim Basin. *Xinjiang Petroleum Geol.* **46**(1), 1–12. 10.7657/XJPG20250101 (2025).

[11] Zhang, J. B. et al. Study on development mechanism and variability of strike-slip fault-controlled reservoirs regulated by multi-stage structural stress: A case study of the shunbei area, Tarim basin. *Petroleum Geol. Exp.* **46**(4), 775–785. 10.11781/sysydz202404775 (2024).

[12] Huang, Y., Zhao, A., Zhang, T. & Guo, W. Plastic failure zone characteristics and stability control technology of roadway in the fault area under non-uniformly high geostress: A case study from Yuandian Coal Mine in Northern Anhui Province, China. *Open Geosci.* **12**(1), 406–424. 10.1515/geo-2020-0154 (2020).

[13] Zhang, Y., Hou, S., Mei, S., Zhao, Y. & Li, D. Finite volume method-based numerical simulation method for hydraulic fracture initiation in rock around a perforation. *J. Zhejiang Univ. Sci. A* **24**(1), 56–63. 10.1631/jzus.a2200203 (2023).

[14] Zhou, Z. et al. Analysis of interaction mechanism between surrounding rock and supporting structures for soft-rock tunnels under high geo-stress. *Acta Geotech.* **18**(9), 4871–4897. 10.1007/s11440-023-01857-w (2023).

[15] Cheng, X. et al. Fast modeling method of multi-attribute 3D geological model and application in high geostress tunnel. *J. Eng. Geol.* **31**(3), 959–967. 10.13544/j.cnki.jeg.2021-0038 (2023).

[16] Li, J. et al. Numerical simulation of the palaeotectonic stress field and prediction of the natural fracture distribution in shale gas reservoirs: A case study in the Longmaxi formation of the Luzhou area, southern Sichuan Basin, China. *Geol. J.* **58**(11), 4165–4180. 10.1002/gj.4744 (2023).

[17] Zhou, Z. Numerical simulation of rock breaking by diamond particles under ultra deep drilling conditions. *Acad. J. Sci. Technol.* **10**(1), 258–266. 10.54097/9nze7p91 (2024).

[18] Zhou, Z., Chen, Z., He, C. & Kou, H. Investigation on the evolution characteristics and transfer mechanism of surrounding rock pressure for a hard-rock tunnel under high geo-stress: Case study on the Erlang mountain tunnel, China. *Bull. Eng. Geol. Environ.* **80**(11), 8339–8361. 10.1007/s10064-021-02439-4 (2021).

[19] Liao, W. et al. Evaluation on the dynamic sealing capacity of underground gas storages rebuilt from gas reservoirs: A case study of Xinjiang H underground gas storage. *Nat. Gas Ind. B* **8**(4), 334–343. 10.1016/j.ngib.2021.07.003 (2021).

[20] Zhao, X., Li, H. & Zhang, S. Analysis of the spalling process of rock mass around a deep underground ramp based on numerical modeling and in-situ observation. *Geomat. Nat. Hazards Risk.* **11**(1), 1619–1637. 10.1080/19475705.2020.1808085 (2020).

[21] Zhou, H. et al. Development and numerical modeling approached to individual rock test chamber based on in-situ condition preserved. *Rock Mech. Rock Eng.* **55**(11), 7049–7062. 10.1007/s00603-022-03019-y (2022).

[22] Tao, Z.-G. et al. Model test on support scheme for carbonaceous slate tunnel in high geostress zone at high depth. *J. Mt. Sci.* **18**(3), 764–778. 10.1007/s11629-020-6509-1 (2021).

[23] Hao, Y. et al. Numerical modeling on strain energy evolution in rock system interaction with energy-absorbing prop and rock bolt. *Int. J. Min. Sci. Technol.* **33**(10), 1273–1288. 10.1016/j.ijmst.2023.08.007 (2023).

[24] Tan, N., Yang, R. & Tan, Z. Influence of complicated faults on the differentiation and accumulation of in-situ stress in deep rock mass. *Int. J. Miner. Metall. Mater.* **30**(5), 791–801. 10.1007/s12613-022-2528-y (2023).

[25] Li, Z., Wang, S., Li, L., Zhang, J. & Li, T. Numerical simulation of brittleness effect on propagation behavior of glutenite hydraulic fractures. *Ain Shams Eng. J.* **12**(4), 3419–3427. 10.1016/j.asej.2021.03.015 (2021).

[26] Ren, S.-L., He, M.-C., Lin, W.-J., Zhang, T.-W. & Tao, Z.-G. Geomechanics model test and numerical simulation of 2G-NPR bolt support effect in an active fault tunnel. *J. Mt. Sci.* **19**(9), 2729–2741. 10.1007/s11629-022-7538-8 (2022).

[27] Dai, Z., Yang, J., Dai, R. & Zhu, Q. Three-dimensional and threefold nonlinear numerical modeling for slope-stabilizing pile. *KSCE J. Civ. Eng.* **26**(11), 4390–4406. 10.1007/s12205-022-1474-6 (2022).

[28] Li, J. et al. Initial geostress in underground cavities obtained by in situ measurements and BP-ANN inversion. *Geotech. Geol. Eng.* **42**(2), 1049–1062. 10.1007/s10706-023-02604-4 (2024).

[29] Wang, M. et al. Analysis of rock mass parameters and plastic zone of a tunnel in southwest mountainous area. *KSCE J. Civ. Eng.* **27**(12), 5448–5459. 10.1007/s12205-023-0886-2 (2023).

[30] Meng, H. et al. Casing deformation mechanisms of horizontal wells in seismically active zones: A comprehensive analysis. *Sci. Rep.* **15**(1), 10746–10761. 10.1038/s41598-025-94469-1 (2025). 40155656 10.1038/s41598-025-94469-1 PMC11953276

[31] Yaghoubi, A., Dusseault, M. B. & Leonenko, Y. Injection-induced fault slip assessment in Montney formation in Western Canada. *Sci. Rep.* **12**(1), 11551–11563. 10.1038/s41598-022-15363-8 (2022). 35798806 10.1038/s41598-022-15363-8 PMC9262911

[32] Zhao, C. J., Jin, Y. X. & Wang, X. Investigation on dynamic mechanism of fault slip and casing deformation during multi-fracturing in shale gas wells. *Sci. Rep.* **14**(1), 13164–13181. 10.1038/s41598-024-63923-x (2024). 38849428 10.1038/s41598-024-63923-x PMC11161626

